# Pneumothorax as a Presenting Clinical Manifestation of Metastatic Prostate Cancer

**DOI:** 10.4021/wjon630w

**Published:** 2013-05-06

**Authors:** Kuo-Hwa Chiang, Shun-Hsing Hung, Sheng-Tsung Chang

**Affiliations:** a Division of Chest Medicine, Department of Internal Medicine, Chi Mei Medical Center, Tainan, Taiwan; b Department of Information Management, Chia Nan University of Pharmacy and Science, Tainan, Taiwan; c Department of Urology, Chi Mei Medical Center, Tainan, Taiwan; d Department of Pathology, Chi Mei Medical Center, Tainan, Taiwan

**Keywords:** Prostate adenocarcinoma, Pneumothorax, Cervical lymph node, Pulmonary metastases

## Abstract

Pulmonary metastases are not encountered commonly in patients with prostate cancer. Pulmonary metastases with pneumothorax as a presenting clinical manifestation in newly diagnosed prostate cancer are very rare. Here, we present the case of an 82-year-old patient who was admitted to our center with a chief complaint of worsening dyspnea over the past few days. The chest X-ray and computed tomography (CT) showed left pneumothorax and bilateral lung opacities as well as generalized lymphadenopathy and diffuse bony metastases. After a series of workup including cervical lymph node biopsy with immunohistochemical staining, abdomen CT, serum prostate-specific antigen (PSA), and transrectal ultrasonography (TRUS), he was proved to have prostate cancer with multiple lung, bone and lymph node metastases. This case is reported because of the rarity for a prostate carcinoma presented clinically with an unusual pulmonary manifestation.

## Introduction

Prostate cancer is the most common noncutaneous human malignancy, with an estimated 241,740 new cases in the USA in 2012. It is also the second-most lethal tumor among men, with 28,170 expected deaths in 2012 [1]. The most common sites of metastases of prostate are regional lymph nodes and bones, followed by lung, bladder, liver, adrenal gland and kidney. Prostate cancer with supraclavicular lymph nodes metastases is rare, with a reported incidence of approximately 0.28% [2]. Pulmonary metastases with pneumothorax as a presenting clinical manifestation in newly diagnosed prostate cancer are very rare. Here, we present an unusual case of 82-year-old male without previous cancer history, who admitted to our hospital due to pneumothorax with collapse of left lung, and after a series of workup he was proved to have prostate cancer with lung and left lower cervical, left supraclavicular, mediastinum and abdomen lymph node and bony metastases. To our knowledge, this is the first case report in English literature.

## Case Report

**Figure 1:**
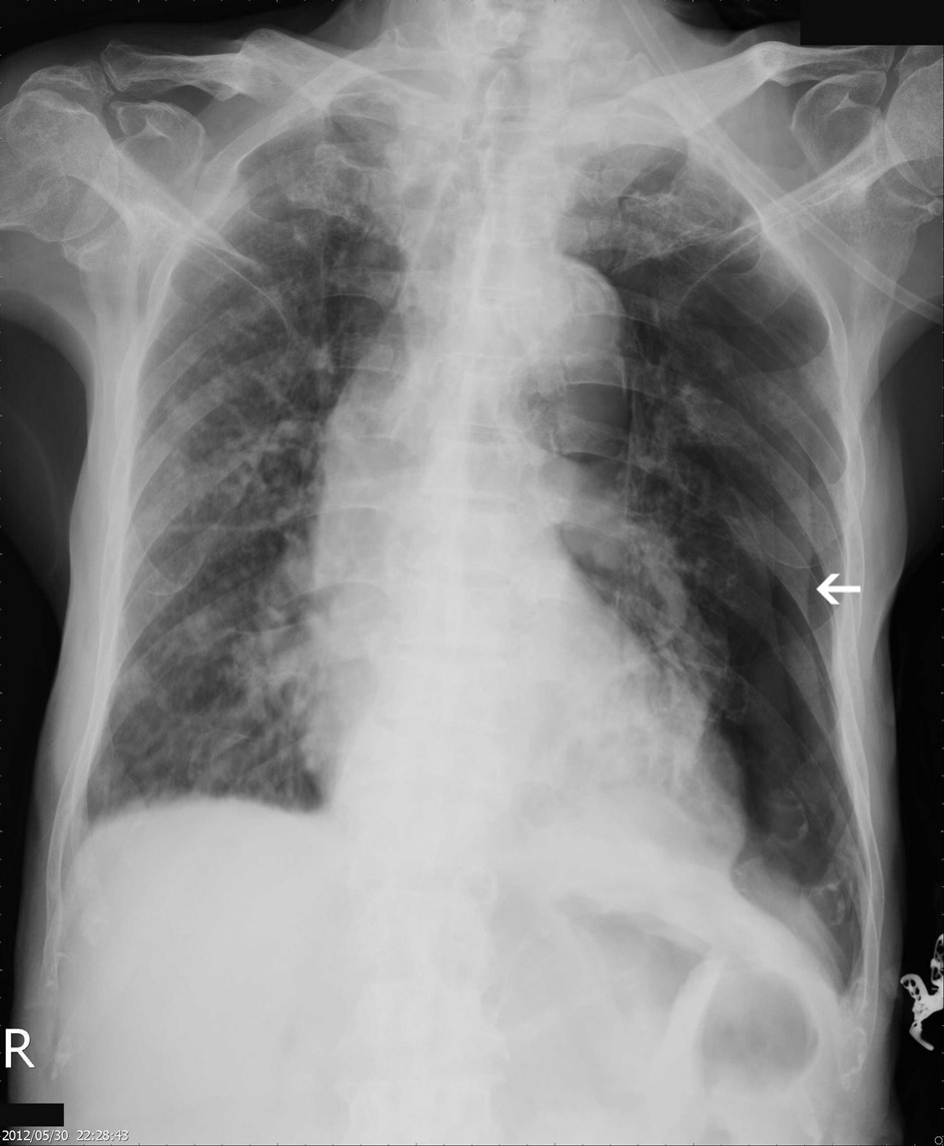
Initial CXR showing left pneumothorax, with ground grass densities over right lung.

**Figure 2:**
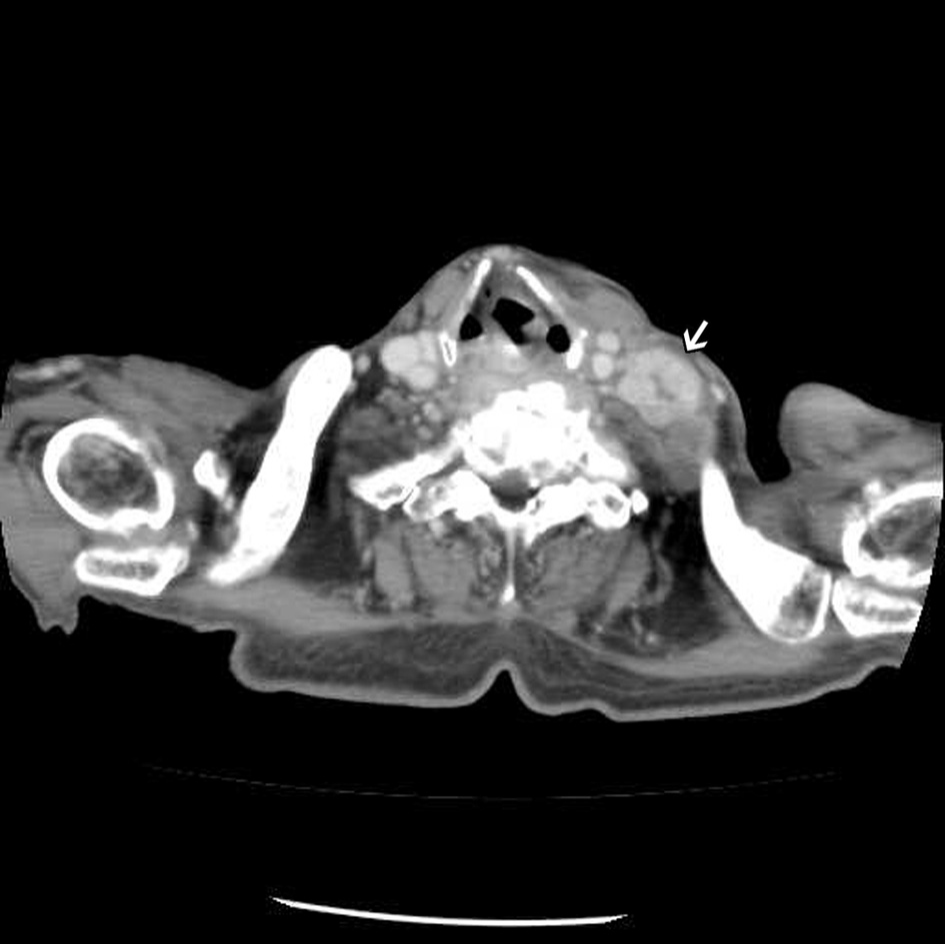
Enhanced chest CT showing an enlarged lymph node at left lower neck.

**Figure 3:**
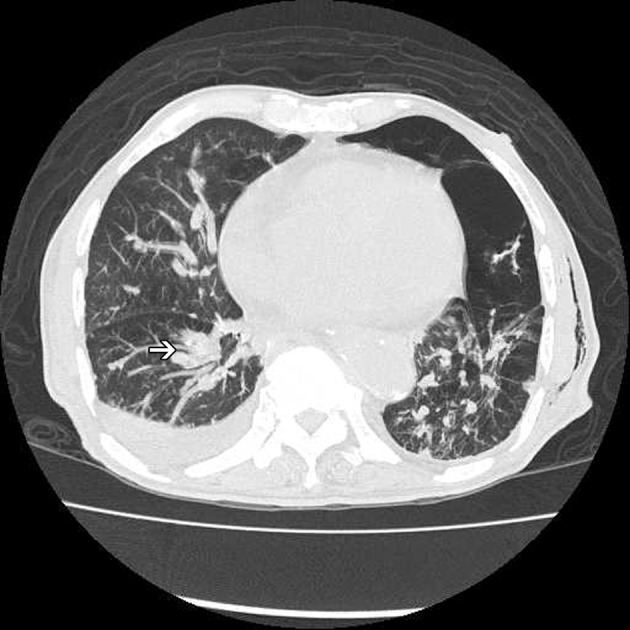
High resolution computed tomography (HRCT) of the chest showing hilar lymphadenopathy (arrow) and diffuse reticular nodular lesions over bilateral lung, with left residule pneumothorax and subcutaneous emphysema over left chest wall.

**Figure 4:**
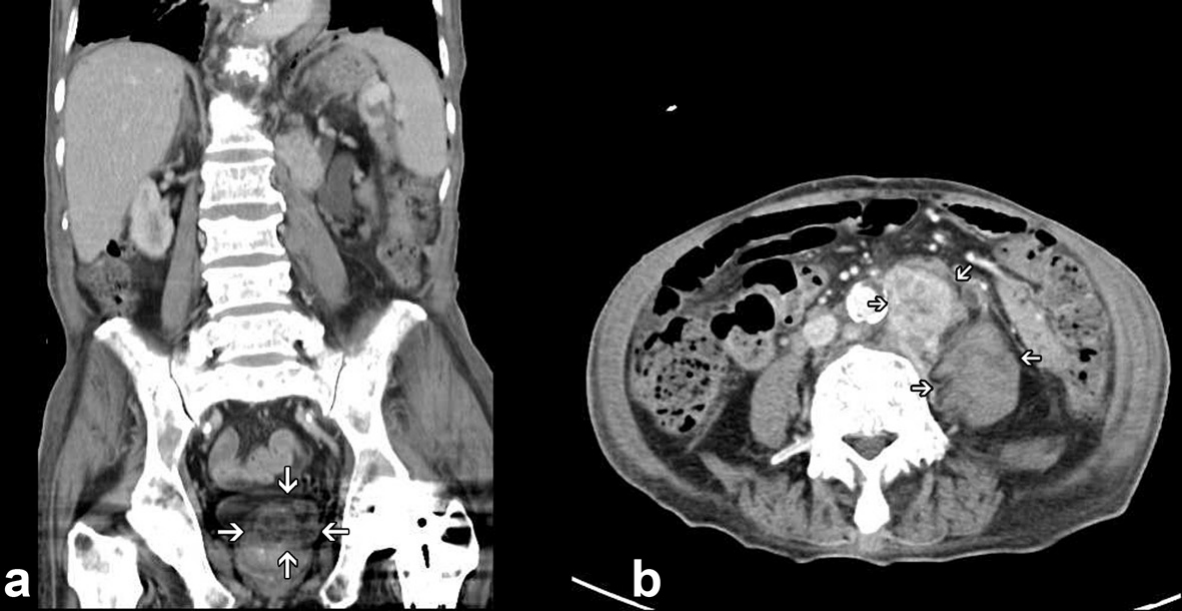
Enhanced lower abdomen CT showing a 4.3 × 3.0 cm ill defined heterogenous mass lesion over left lobe of prostate (a); with extensive para-aortic and retroperitoneal lymphadenopathy (b).

**Figure 5:**
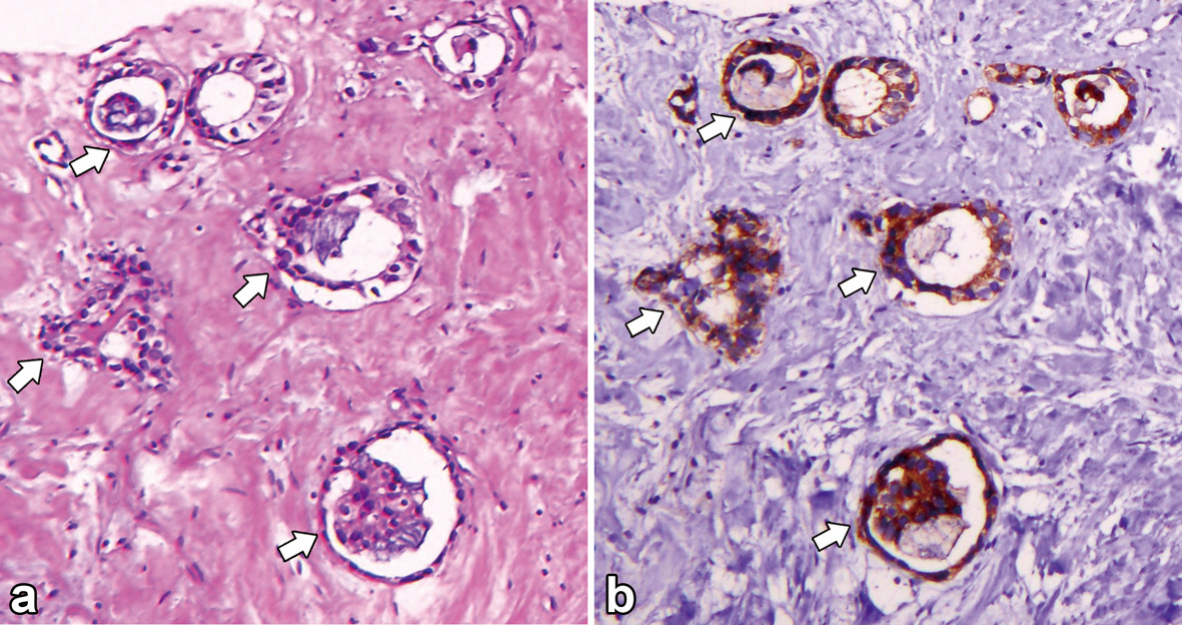
(a) Hematoxyline and Eosin (H&E) stain of biopsy specimen of the left lower neck lymph node showing tubuloglandular structures with stromal fibrosis; (b) Immunohistochemical stain with P504s showing neoplastic cells express P504s diffusely.

An 82-year-old male patient presented with chest tightness and shortness of breathing for 10 days. His past medical history included hypertension and old pulmonary tuberculosis. He suffered from productive cough for weeks and was referred to our center because of the worsening symptoms of dyspnea and lower leg edema noted over the past few days. On presentation, his consciousness was alert. Body temperature was 37.8 °C. Pulse was 136 per minute regular. Blood pressure was 164/90 mmHg. Oxygen saturation was 88%. Auscultation revealed decreased breathing sounds at left lung field and sonorous rhonchus at right lung field. X-ray of the chest showed left pneumothorax with collapse of left lung, and ground glass opacities in right lung field (Fig. 1), more in favor of inflammatory process at that time. He was admitted under the impression of right lung pneumonia with left pneumothorax and received chest tube insertion and empiric antibiotics treatment. However persistent air leak noted, he then received a chest CT for further survey. A chest CT showed nodular lesions over bilateral lung, with diffuse bone, left lower neck (Fig. 2), left suparclavicular, mediastinum, as well as right hilar (Fig. 3) and lower abdominal lymph nodes metastases. Unknown primary malignancy with bilateral lung metastases, diffuse bony metastases with left supraclavicular and left neck lymph node metastases was impressed and further tissue proof for possible underlying malignancy was recommended. For this reason, an echo guided biopsy with left neck lymph node biopsy was arranged for further study because the surgical intervention was not recommended for the patient due to poor lung function while patient declined bronchoscopy biopsy either. A subsequent neck lymph node biopsy revealed metastatic adenocarcinoma, while immunohistochemical stain for thyroid transcription factor -1(TTF-1), and cytokeratin 20 (CK20) are all negative. Serum tumor markers were examined in an attempt to identify the primary lesion, revealing a PSA level of > 1,000 ng/mL (normal < 4.0 ng/mL) while CEA 2.7 ng/mL (normal < 5.0 ng/mL). Digital rectal examination showed an enlarged and hardened left prostate lobe. An abdomen CT disclosed 4.3 × 3.0 cm ill defined heterogenous mass lesion at lower lobe of prostate (Fig. 4a) with regional and non-regional lymph nodes (Fig. 4b) and bony metastases. The urologist was consulted, and transrectal ultrasonography (TRUS) showed irregular urinary bladder wall thickening with enlargement of prostate, hypoechoic nodule with increased vascularity in left peripheral zone of prostate. Meanwhile the patient refused further tissue proof due to his poor general condition. The pathologist was informed of the elevated serum PSA and further clinical data, then an additional immunohistochemical staining of the left neck lymph node biopsy with P504S was performed as was compared to the regular H&E stain (Fig. 5a), the neoplastic cells expressed P504S diffusely (Fig. 5b), a metastatic adenocarcinoma of prostatic origin was confirmed. However his condition went downhill rapidly, and after thoroughly discussion with the patient and his family, they decided to receive hospice care and refused further intervention.

## Discussion

This case is unique because it presents pneumothorax as the initial manifestation of prostate cancer with generalized lymphadenopathy and lung metastases. This atypical presentation has made diagnosing prostate adenocarcinoma extremely difficult.

Prostate cancer metastases to mediastinal, supraclavicular, and cervical lymph nodes are uncommon [3]. The incidence of cervical lymph node involvement in patients with prostate cancer has been reported as 0.28%; however, these patients almost uniformly present with widespread metastatic disease [4-7]. In this case, the history and the symptoms signs the patient presented didn’t offer much that would help guide the clinician towards the correct primary site of disease. His initial presentations of pneumothorax along with left neck lymphadenopathy once lead our attention to primary lung malignancy. However, after his cervical lymph node biopsy revealed metastatic adenocarcinoma, with a negative TTF1, further series of workup for unknown primary including abdominal CT, transrectal ultrasonography (TRUS)and serum PSA as well as special immunohistochemistry stain of the pathology specimen with P504S had performed. P504S is the first gene identified from prostate cancer by cDNA microarrays to be suitable for clinical practice and to potentially improve the diagnosis of prostate cancer [8]. In distinction to PSA, P504S is highly selective for prostate cancer. A number of studies from several institutions have demonstrated that P504S is an important positive tissue marker for prostate carcinoma regardless of tumor grade [8, 9]. In this case, imaging studies, immunohistochemical stains, and pathological specimens all pointed to a narrow differential, serum tumor markers were obtained to further help in the diagnosis of metastatic prostate cancer. Pulmonary metastases are common in advanced prostate carcinoma, with autopsy studies showing an incidence of 25% to 50% [10] but are rare at diagnosis. Pulmonary manifestations are clinically recognized in only 5% of cases. Metastatic prostate cancer with pneumothorax is extremely rare. The exact mechanism is not clear, but it might be possibly related to extensive lung metastases from prostate cancer. Pulmonary metastases are seen concomitant with lymph node and bone metastases and are most commonly encountered with multi-organ involvement [10, 11]. Multiple spread of carcinoma to the lung is a form of the terminal phase of carcinoma and leads to a poor prognosis. It may have progressed considerably when multiple lesions were observed in lungs, and may follow a miserable course as described in the present case.

The case is an aggressive prostate cancer with a very unusual presentation. Although metastases to the cervical lymph nodes are rare in prostate cancer patients, they should be considered as one of the differential diagnoses of cervical lymphadenopathy in elderly males with adenocarcinoma of undetermined origin even in an atypical presentation. Serum PSA level and subsequent immunohistochemical stains of their lymph node biopsies can be helpful in establishing a definitive diagnosis of prostate cancer.
